# Supplementary material for: Genomic Features of Antimicrobial Resistance in Staphylococcus pseudintermedius Isolated from Dogs with Pyoderma in Argentina and the United States: A Comparative Study
Source: Int J Mol Sci. 2023 Jul 12;24(14):11361. doi: 10.3390/ijms241411361 (PMC10379401; doi:10.3390/ijms241411361)
Supplement: Supplementary file 1 [file ijms-24-11361-s001.zip › ijms-2467064-supplementary.pdf]

Table S1. Best match predictions (highlighted in grey) based on mapping against all SCC*mec* type references.

Isolate ID	Reference (accession number)	ID %	Coverage %	Best match
BI1985	SCC*mec* III (AM904732.1)	99.96	92.60	SCC*mec* III
NY14853PPY10094	SCC*mec* III (AM904732.1)	99.80	100	SCC*mec* III
MI48910PPY10092	SCC*mec* III (AM904732.1)	92.80	100	SCC*mec* III
AL36849PPY10048	SCC*mec* IVa (AB063172)	78.20	76.70	SCC*mec* IVg
	SCC*mec* IVb (AB063173)	78.70	90.60	
	SCC*mec* IVc (AB096217)	83.00	65.50	
	SCC*mec* IVd (AB097677)	87.50	62.00	
	SCC*mec* IVg (DQ106887)	87.30	99.10	
	SCC*mec* IVh (HE681097)	79.80	78.40	
	SCC*mec* IVi (AB425823)	92.10	10.10	
	SCC*mec* IVj (AB425824)	79.60	61.10	
	SCC*mec* IVk (GU122149)	76.20	7.40	
	SCC*mec* IVl (AB633329)	80.30	54.40	
	SCC*mec* IVm (AB872254)	79.60	61.10	
	SCC*mec* IVn (KX385846.1)	82.20	80.30	
AL36849PPY10061	SCC*mec* IVa (AB063172)	84.50	87.30	SCC*mec* IVg
	SCC*mec* IVb (AB063173)	86.10	99.90	
	SCC*mec* IVc (AB096217)	83.10	75.50	
	SCC*mec* IVd (AB097677)	75.70	81.30	
	SCC*mec* IVg (DQ106887)	99.40	99.90	
	SCC*mec* IVh (HE681097)	86.40	81.70	
	SCC*mec* IVi (AB425823)	81.80	94.50	
	SCC*mec* IVj (AB425824)	87.00	95.90	
	SCC*mec* IVk (GU122149)	77.60	41.20	
	SCC*mec* IVl (AB633329)	86.30	62.00	
	SCC*mec* IVm (AB872254)	80.80	23.20	
	SCC*mec* IVn (KX385846.1)	80.70	79.70	
KS66506PPY30089	SCC*mec* IVa (AB063172)	94.80	65.90	SCC*mec* IVg
	SCC*mec* IVb (AB063173)	95.10	77.60	
	SCC*mec* IVc (AB096217)	97.30	52.60	
	SCC*mec* IVd (AB097677)	97.20	52.10	
	SCC*mec* IVg (DQ106887)	99.40	86.20	
	SCC*mec* IVh (HE681097)	97.20	63.70	
	SCC*mec* IVi (AB425823)	91.70	72.70	
	SCC*mec* IVj (AB425824)	98.10	72.00	
	SCC*mec* IVk (GU122149)	94.60	32.30	
	SCC*mec* IVl (AB633329)	97.10	46.60	
	SCC*mec* IVm (AB872254)	92.10	57.10	
	SCC*mec* IVn (KX385846.1)	95.70	59.80	
ND58102PPY20076	SCC*mec* IVa (AB063172)	79.70	69.70	SCC*mec* IVg

	SCC*mec* IVb (AB063173)	80.20	82.20	
	SCC*mec* IVc (AB096217)	97.40	36.10	
	SCC*mec* IVd (AB097677)	96.20	52.70	
	SCC*mec* IVg (DQ106887)	85.90	90.50	
	SCC*mec* IVh (HE681097)	95.90	48.40	
	SCC*mec* IVi (AB425823)	79.20	78.30	
	SCC*mec* IVj (AB425824)	82.50	76.30	
	SCC*mec* IVk (GU122149)	95.00	25.10	
	SCC*mec* IVl (AB633329)	81.80	49.30	
	SCC*mec* IVm (AB872254)	80.00	59.50	
	SCC*mec* IVn (KX385846.1)	94.30	46.60	
BI1979	SCC*mec* Va (AB121219.1)	87.30	52.60	SCC*mec* V (5C2&5)
	SCC*mec* Vb (AB462393.1)	91.50	62.80	
	SCC*mec* Vb (AB512767.1)	91.90	72.60	
	SCC*mec* Vb (AB478780.1)	91.60	72.70	
	SCC*mec* Vc (AB505629.1)	92.50	59.60	
	SCC*mec* V(T) (FJ544922.1)	99.80	95.40	
BI1980	SCC*mec* Va (AB121219.1)	81.30	62.60	SCC*mec* V (5C2&5)
	SCC*mec* Vb (AB462393.1)	83.70	51.20	
	SCC*mec* Vb (AB512767.1)	83.90	59.10	
	SCC*mec* Vb (AB478780.1)	83.70	59.30	
	SCC*mec* Vc (AB505629.1)	82.90	50.60	
	SCC*mec* V(T) (FJ544922.1)	84.80	89.30	
BI1981	SCC*mec* Va (AB121219.1)	84.80	55.20	SCC*mec* V (5C2&5)
	SCC*mec* Vb (AB462393.1)	97.90	47.80	
	SCC*mec* Vb (AB512767.1)	98.50	55.20	
	SCC*mec* Vb (AB478780.1)	98.00	55.30	
	SCC*mec* Vc (AB505629.1)	99.00	49.30	
	SCC*mec* V(T) (FJ544922.1)	99.70	91.30	
BI1984	SCC*mec* Va (AB121219.1)	80.00	54.80	SCC*mec* V (5C2&5)
	SCC*mec* Vb (AB462393.1)	89.00	42.90	
	SCC*mec* Vb (AB512767.1)	89.30	49.40	
	SCC*mec* Vb (AB478780.1)	89.00	49.60	
	SCC*mec* Vc (AB505629.1)	88.50	43.10	
	SCC*mec* V(T) (FJ544922.1)	91.50	71.80	
BI1990	SCC*mec* Va (AB121219.1)	81.70	54.80	SCC*mec* V (5C2&5)
	SCC*mec* Vb (AB462393.1)	90.20	42.10	
	SCC*mec* Vb (AB512767.1)	90.10	50.40	
	SCC*mec* Vb (AB478780.1)	90.20	48.80	
	SCC*mec* Vc (AB505629.1)	91.10	38.90	
	SCC*mec* V(T) (FJ544922.1)	91.60	65.50	
BI1991	SCC*mec* Va (AB121219.1)	82.90	48.10	SCC*mec* V (5C2)
	SCC*mec* Vb (AB462393.1)	83.80	28.60	
	SCC*mec* Vb (AB512767.1)	83.80	33.40	

	SCC*mec* Vb (AB478780.1)	83.40	33.00	
	SCC*mec* Vc (AB505629.1)	85.10	27.90	
	SCC*mec* V(T) (FJ544922.1)	73.00	61.30	
BI2002	SCC*mec* Va (AB121219.1)	79.40	69.00	SCC*mec* V (5C2)
	SCC*mec* Vb (AB462393.1)	80.10	39.60	
	SCC*mec* Vb (AB512767.1)	80.10	46.30	
	SCC*mec* Vb (AB478780.1)	80.00	45.80	
	SCC*mec* Vc (AB505629.1)	84.80	28.40	
	SCC*mec* V(T) (FJ544922.1)	73.30	62.20	
BI2003	SCC*mec* Va (AB121219.1)	81.70	56.60	SCC*mec* V (5C2&5)
	SCC*mec* Vb (AB462393.1)	87.50	54.00	
	SCC*mec* Vb (AB512767.1)	87.80	62.40	
	SCC*mec* Vb (AB478780.1)	87.50	62.50	
	SCC*mec* Vc (AB505629.1)	93.70	38.90	
	SCC*mec* V(T) (FJ544922.1)	94.70	72.50	
BI2004	SCC*mec* Va (AB121219.1)	79.90	49.10	SCC*mec* V (5C2&5)
	SCC*mec* Vb (AB462393.1)	91.90	48.50	
	SCC*mec* Vb (AB512767.1)	92.30	56.00	
	SCCmec Vb (AB478780.1)	92.70	54.20	
	SCC*mec* Vc (AB505629.1)	92.20	44.40	
	SCC*mec* V(T) (FJ544922.1)	93.50	82.90	
BI2008	SCC*mec* Va (AB121219.1)	90.60	37.90	SCC*mec* V (5C2)
	SCC*mec* Vb (AB462393.1)	88.10	24.70	
	SCC*mec* Vb (AB512767.1)	87.30	30.30	
	SCC*mec* Vb (AB478780.1)	88.10	28.60	
	SCC*mec* Vc (AB505629.1)	88.50	24.90	
	SCCmec V(T) (FJ544922.1)	75.70	55.80	
KY40511PPY30123	SCC*mec* Va (AB121219.1)	81.40	42.40	SCC*mec* V (5C2&5)
	SCC*mec* Vb (AB462393.1)	86.50	54.60	
	SCC*mec* Vb (AB512767.1)	87.60	61.80	
	SCC*mec* Vb (AB478780.1)	87.20	62.10	
	SCC*mec* Vc (AB505629.1)	90.50	42.30	
	SCC*mec* V(T) (FJ544922.1)	92.00	85.10	
LA70803PPY30016	SCC*mec* Va (AB121219.1)	70.90	28.90	SCC*mec* V (C52)
	SCC*mec* Vb (AB462393.1)	49.80	33.20	
	SCC*mec* Vb (AB512767.1)	50.10	38.10	
	SCC*mec* Vb (AB478780.1)	49.80	38.50	
	SCC*mec* Vc (AB505629.1)	50.00	34.10	
	SCCmec V(T) (FJ544922.1)	50.00	57.80	
MI48910PPY20187	SCC*mec* Va (AB121219.1)	87.10	37.70	SCC*mec* V (5C2&5)
	SCC*mec* Vb (AB462393.1)	84.40	45.30	
	SCC*mec* Vb (AB512767.1)	84.90	51.80	
	SCC*mec* Vb (AB478780.1)	84.60	52.20	
	SCC*mec* Vc (AB505629.1)	84.90	39.30	

	SCC*mec* V(T) (FJ544922.1)	86.20	74.10	
MO65211PPY31146	SCC*mec* Va (AB121219.1)	32.20	100.00	SCC*mec* V (5C2&5)
	SCC*mec* Vb (AB462393.1)	45.60	100.00	
	SCC*mec* Vb (AB512767.1)	28.40	100.00	
	SCC*mec* Vb (AB478780.1)	48.00	100.00	
	SCC*mec* Vc (AB505629.1)	45.40	100.00	
	SCC*mec* V(T) (FJ544922.1)	99.99	100.00	
TX77840PPY20121	SCC*mec* Va (AB121219.1)	84.90	70.00	SCC*mec* V (5C2&5)
	SCC*mec* Vb (AB462393.1)	94.70	56.80	
	SCC*mec* Vb (AB512767.1)	95.40	65.70	
	SCC*mec* Vb (AB478780.1)	94.80	65.80	
	SCC*mec* Vc (AB505629.1)	98.30	53.80	
	SCC*mec* V(T) (FJ544922.1)	99.98	80.20	
WI53706PPY30014	SCC*mec* Va (AB121219.1)	69.10	100.00	SCC*mec* V (5C2&5)
	SCC*mec* Vb (AB462393.1)	46.30	99.50	
	SCC*mec* Vb (AB512767.1)	48.90	99.40	
	SCC*mec* Vb (AB478780.1)	48.80	99.40	
	SCC*mec* Vc (AB505629.1)	48.50	100.00	
	SCC*mec* V(T) (FJ544922.1)	52.10	86.10	
